# Alteration of Hyaluronic Acid Metabolism in Tumor Microenvironment Can Modulate DNA Repair Gene Expression: Therapeutic Potential for Triple-Negative Breast Cancer

**DOI:** 10.3390/ijms262311328

**Published:** 2025-11-24

**Authors:** Ina Sevic, Daiana Lujan Vitale, Candela Moran Maidana, Paolo Rosales, Antonella Icardi, Catalina Latina, Lucia Romano, Alejandra Brandone, Paula Giannoni, Laura Alaniz

**Affiliations:** 1 Laboratorio de Microambiente Tumoral, Centro de Investigaciones Básicas y Aplicadas (CIBA), Centro de Investigaciones y Transferencia del Noroeste de la Provincia de Buenos Aires (CIT NOBA), Universidad Nacional del Noroeste de la Provincia de Buenos Aires, Consejo Nacional de Investigaciones Científicas y Técnicas (UNNOBA-UNSAdA-CONICET), Junín B6000, Argentina; 2 Centro de Investigaciones Básicas y Aplicadas (CIBA), Centro de Investigaciones y Transferencia del Noroeste de la Provincia de Buenos Aires (CIT NOBA), Universidad Nacional del Noroeste de la Provincia de Buenos Aires, Consejo Nacional de Investigaciones Científicas y Técnicas (UNNOBA-UNSAdA-CONICET), Junín B6000, Argentina; 3 Hospital Interzonal General de Agudos “Dr. Abraham Felix Piñeyro”, Junín B6000, Argentina; 4 Clínica Centro, Junín B6000, Argentina

**Keywords:** tumor microenvironment, BRCA1, BRCA2, hyaluronan metabolism, breast cancer, colorectal cancer

## Abstract

Breast and colorectal cancers remain among the leading causes of cancer-related deaths globally, with therapy failure often driven by tumor complexity and interactions with the tumor microenvironment (TME). Hyaluronic acid (HA), a key extracellular matrix component, plays a vital role in TME remodeling, while altered breast cancer gene 1 and 2 (*BRCA1/2*) expression, essential for DNA repair, is linked to cancer aggressiveness. This study investigates the link between HA metabolism and BRCA1/2 expression in breast and colorectal cancers. We analyzed HA, CD44, and BRCA1 and 2 expression in patient tissue samples via immunofluorescence. To assess whether HA metabolism affects BRCA1/2 expression, we treated spheroids with hyaluronidase (HYAL) and 4-methylumbelliferone (4-MU) to reduce HA levels. The resulting changes in BRCA1/2 expression were evaluated using qPCR, and tumor profiles were assessed through microscopy and immunofluorescence. We found a coordinated behavior between BRCA1 and BRCA2 in breast cancer and observed BRCA1’s crypt-restricted expression in normal colorectal tissue, which may underlie its well-known tissue specificity. In a triple-negative breast cancer model, we observed that 4-MU reduced spheroid volume and increased BRCA 1/2 levels, suggesting a potential mechanism of 4-MU for tumor shrinkage and BRCA restoration. These findings suggest that 4-MU, a compound already approved for oral use in hepatobiliary indications in Europe and Asia, is a mechanistically plausible HA-targeting candidate for therapeutic repurposing in BRCA-deficient tumors.

## 1. Introduction

Breast cancer is one of the five leading causes of cancer death worldwide [1], while colorectal cancer still ranks as the third most prevalent and second most lethal cancer worldwide [2]. Crucial problems in cancer management are late diagnosis and treatment failure. One of the main reasons for therapy failure is tumor complexity, which depends on the intrinsic characteristics of the tumor cells on the one hand and the tumor microenvironment (TME) and their interplay on the other hand. The TME has a very important role in tumor development and progression that depends on changes in its composition that occur in time [1]. These dynamic alterations in TME can lead to metabolic reprogramming and changes in transcription and signaling networks, which can promote tumorigenesis and in turn result in changes in global genetic expression [3,4]. The TME is composed of an extracellular matrix (ECM) and different non-tumoral cells, like stromal or immune cells [1]. One of the ECM molecules recognized as a key regulator of TME in tumor development is glicosaminoglican hyaluronic acid (HA). HA plays an important role in the organization of tissue architecture and regulation of cell processes like apoptosis, proliferation, invasion, and multidrug resistence [5]. The concentration, molecular weight, and distribution of HA, and, as a consequence, its role, depend on many factors, including tissue type and disease progression, and are regulated mostly by the balace in its synthesis and degradation. There are three HA synthases (HAS1, HAS2, and HAS3) and four hyaluronidases (HYAL1, HYAL2, HYAL3, and HYAL4) involved in HA methabolism [6]. HAS2 is responsible for a major portion of HA synthesis, while HYAL1 and HYAL2 are responsible for major portion of degradation. Transmembrane protein Cluster of differentiation 44 (CD44) is considered the main HA receptor, and their interaction can control different cellular functions like adhesion and migration which can, in turn, influence tumor development [7]. 4-methylumbelliferone (4-MU), a coumarin derivative that inhibits hyaluronan synthesis, is gaining attention for its potential therapeutic use in inflammation and cancer. It has been reported that 4-MU can inhibit HA production in multiple cell lines and tissue types both in vitro and in vivo [1,8,9].

Breast cancer genes (*BRCA*) 1 and 2, are tumor suppressor genes whose protein products are critical for genome integrity preservation by participating in the homologous recombination repair mechanism that repairs DNA double-strand breaks [10,11]. On the other hand, BRCA1 and BRCA2 also protect stalled DNA replication forks from degradation, and in this manner control genomic instability throughout DNA replication [12,13]. BRCA1 and 2 are expressed ubiquitously and are recognized for their tumor suppressor role, with somewhat perplexing tissue specificity (principaly breast and ovarian) [14].

The aim of this study was to investigate whether the TME, specifically HA and its metabolism members, could influence the expression of tumor supressor genes *BRCA1* and *2*, thereby contributing to tumorigenic processes in breast and colorectal cancer. By analyzing both patient-derived tumor samples and established cancer cell lines, we aim to bridge clinical relevance with controlled experimental modeling, providing a comprehensive understanding of how HA metabolism may modulate cancer progression.

## 2. Results

### 2.1. HA and CD44 Tissue Analysis

**Figure 1:**
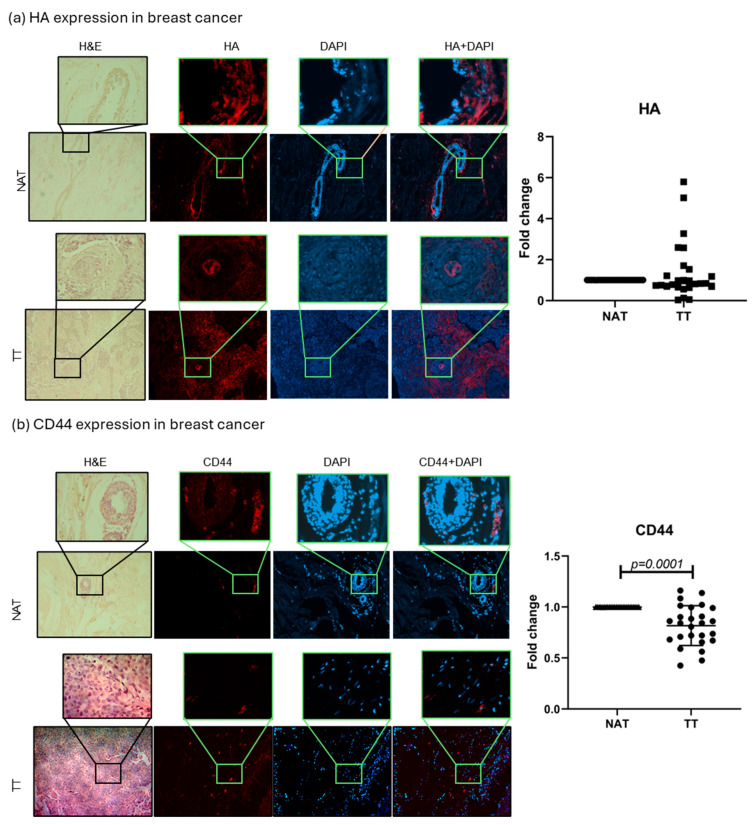
HA and CD44 breast cancer tissue analysis. Representative images of HA (**a**) and CD44 (**b**) protein expression in TT and NAT in breast cancer. Images are shown at 100× and selections at 400× magnification. Graphs (right) show fold change. *p*-values lower than 0.05 were considered significant.

**Figure 2:**
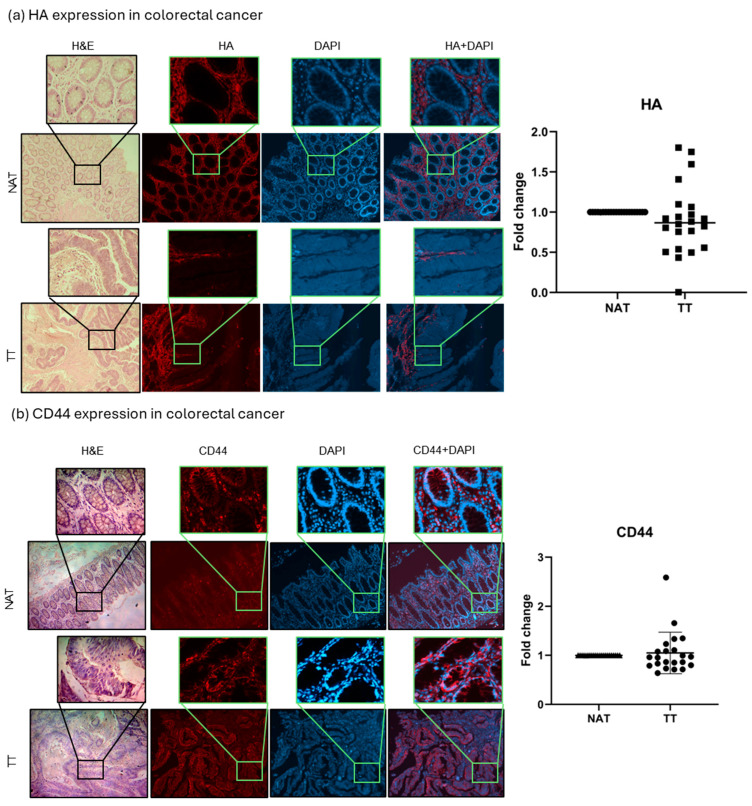
HA and CD44 colorectal cancer tissue analysis. Representative images of HA (**a**) and CD44 (**b**) protein expression in TT and NAT in colorectal cancer. Images are shown at 100× and selections at 400× magnification. Graphs (right) show fold change. *p*-values lower than 0.05 were considered significant.

HA and CD44, the main HA receptor, levels were evaluated in tumor tissue (TT) and non-tumor tissue adjacent to the tumors (NAT) of both breast and colorectal cancer patients. The results are presented as fold change values calculated as TT over matched NAT. There was no significant difference in the HA expression between TT and NAT in breast cancer nor in colorectal cancer patients (1.383- and 0.9039-fold change, respectively; Figure 1 and Figure 2). This could indicate that HA is not differentially expressed in tumors compared to histologically normal tissue; hence, we wondered if it could be differentially expressed between different tumor stages (T1, T2, T3, T4) or tumor cell profiles (proliferation rate, Nottingham grade, hormone receptor expression). A significantly higher HA expression was found in T2 colorectal cancer when compared to T3 and T4 (*p* = 0.021; median fold change values: T2 = 1.716, T3 = 0.7844, T4 = 0.7957; Supplementary Figure S3), while no other comparison had significant results. On the other hand, when comparing CD44 expression, a lower CD44 expression was found in the TT of breast cancer compared to NAT (0.8175-fold change, *p* = 0.0001, Figure 1), while there were no significant differences in colorectal cancer (1.049-fold change, Figure 2).

### 2.2. BRCA1 and BRCA2 Tissue Analysis

**Figure 3:**
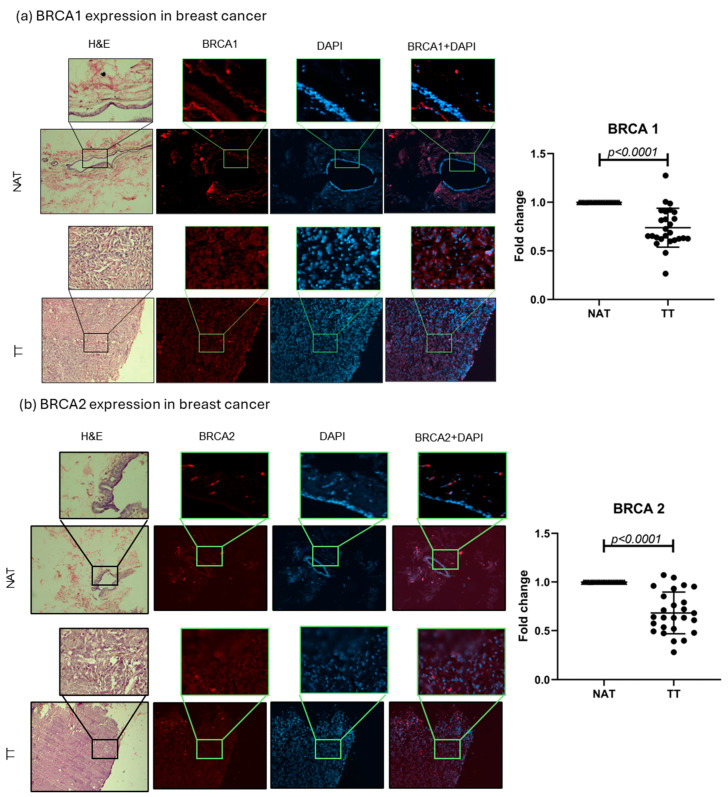
BRCA1 and 2 breast cancer tissue analysis. Representative images of BRCA1 and 2 protein expression in TT and NAT in breast cancer (**a**,**b**). Images are shown at 100× and selections at 400× magnification. Graphs (right) show fold change. *p*-values lower than 0.05 were considered significant.

**Figure 4:**
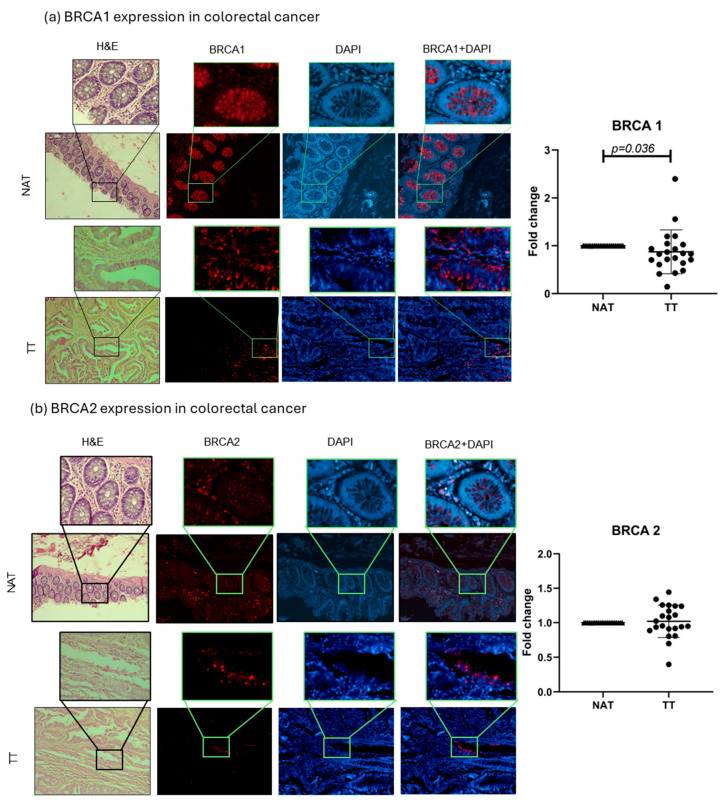
BRCA1 and 2 colorectal cancer tissue analysis. Representative images of BRCA1 and 2 protein expressions in TT and NAT in colorectal cancer (**a**,**b**). Images are shown at 100× and selections at 400× magnification. Graphs (right) show fold change. *p*-values lower than 0.05 were considered significant.

BRCA1 and 2 protein expressions were evaluated in TT and NAT in breast and colorectal cancer. The results are presented as fold change values calculated as TT over matched NAT. In breast cancer, both BRCA1 and BRCA2 levels were significantly lower in TT compared with NAT (0.7378-fold change, *p* < 0.0001, and 0.6822-fold change, *p* < 0.0001, respectively; Figure 3). In colorectal cancer, only BRCA1 expression was significantly lower in TT (0.8740-fold change, *p* = 0.036), while BRCA2 expression showed no significant difference (1.021-fold change; Figure 4). No differences in expression were observed when separated by different tumor stages or tumor cell profiles (Supplementary Figure S3). Interestingly, the majority of the observed BRCA1 expression in healthy colorectal tissue is localized in the crypt area, while *lamina propria* showed very little expression. This was also observed in tumor tissue even though the architecture is changed by the abnormal cell growth. BRCA2 showed no such histological specificity (Figure 4).

### 2.3. Correlation Analysis

**Figure 5:**
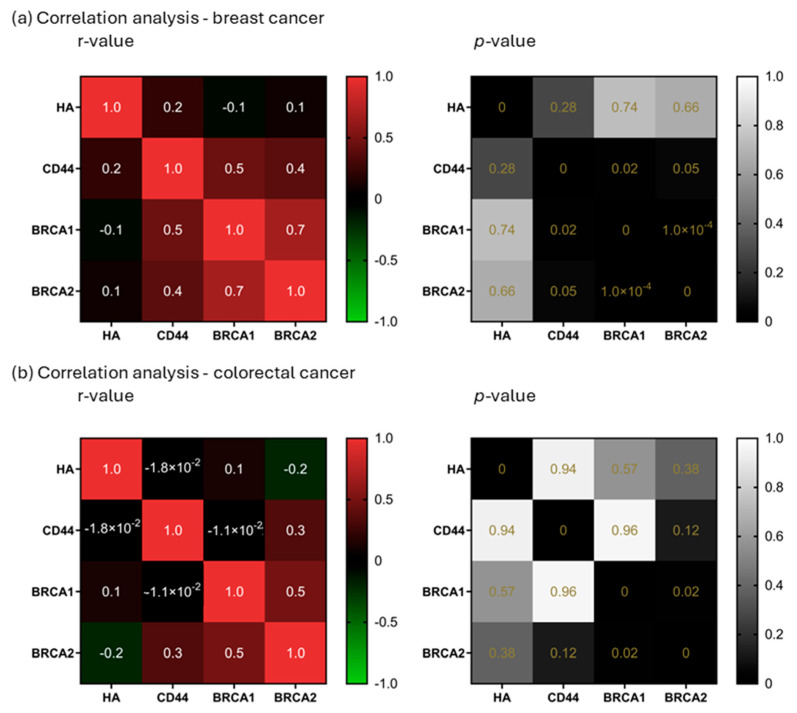
Correlation analysis. Correlation analysis of HA, CD44, BRCA1, and BRCA2 in breast (**a**) and colorectal cancer (**b**). r-values ≥ 0.7 (or −0.7 for negative relationships) were considered a strong correlation (left). *p*-values lower than 0.05 were considered significant (right).

The correlation analysis of HA, CD44, BRCA1, and BRCA2 showed only one strong correlation, BRCA1 and BRCA2 in breast cancer (r = 0.7; *p* < 0.0001), while in the case of colorectal cancer this correlation was much weaker (r = 0.5; *p* = 0.02). Another weak correlation was found in the case of breast cancer, between CD44 and BRCA1 (r = 0.5; *p* = 0.02). Weak correlations are a common appearance in the patient’s results, especially if the number of patients is relatively small, which is why they should be tested in a bigger cohort in order to assess r-values in a more realistic manner (Figure 5).

### 2.4. Spheroid Volume and mRNA Expression Analysis

**Figure 6:**
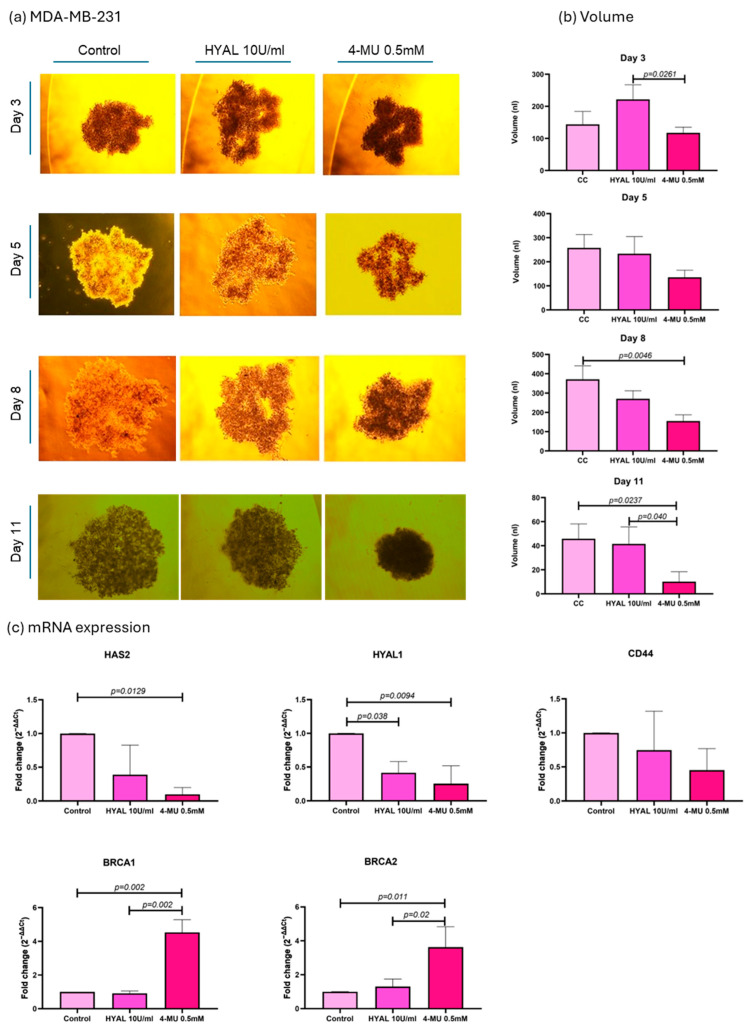
MDA-MB-231 cell line analysis. Representative images of spheroid volume measured at 3, 5, 8, and 11 days (**a**) and statistical analysis (**b**) are shown for control (CC), hyaluronidase (HYAL), and 4-metilumbeliferone (4-MU) treatment. mRNA expression of HAS2, CD44, HYAL1, and BRCA1 and 2 (**c**). *p*-values lower than 0.05 were considered significant. Only *p*-values indicating statistical significance are shown.

**Figure 7:**
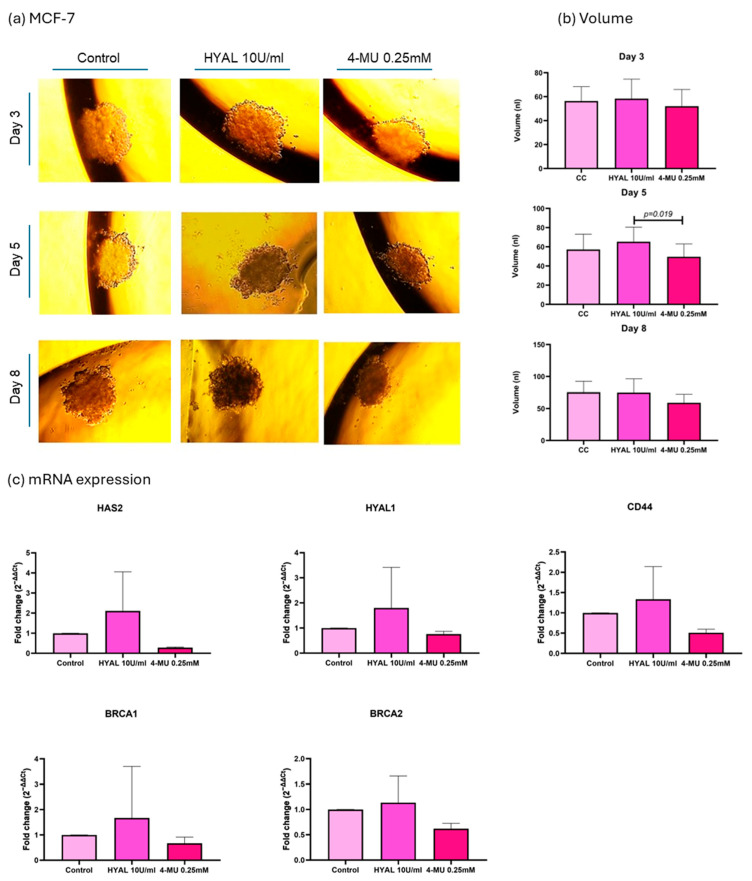
MCF-7 cell line analysis. Representative images of spheroid volume measured at 3, 5, and 8 days (**a**) and statistical analysis (**b**) are shown for control (CC), hyaluronidase (HYAL), and 4-metilumbeliferone (4-MU) treatment. mRNA expression of HAS2, CD44, HYAL1, and BRCA1 and 2 (**c**). *p*-values lower than 0.05 were considered significant. Only *p*-values indicating statistical significance are shown.

**Figure 8:**
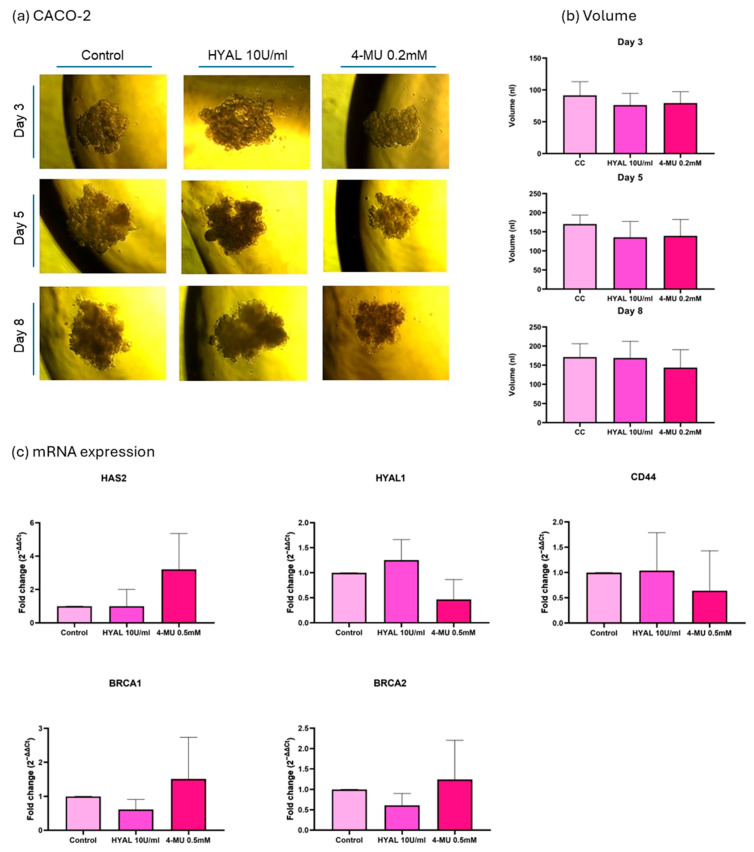
CACO-2 cell line analysis. Representative images of spheroid volume measured at 3, 5, and 8 days (**a**) and statistical analysis (**b**) are shown for control (CC), hyaluronidase (HYAL), and 4-metilumbeliferone (4-MU) treatment. mRNA expression of HAS2, CD44, HYAL1, and BRCA1 and 2 (**c**). *p*-values lower than 0.05 were considered significant. Only *p*-values indicating statistical significance are shown.

As the aim of our experiments was to decrease the HA levels in spheroids in order to assess their influence on DNA repair genes, two treatments were applied, HYAL to degrade HA and 4-MU to prevent its synthesis [8]. First, HA’s influence on spheroid volume was evaluated, and the volume was expressed in nanoliters (nl). In the MDA-MB-231 cell line, 4-MU-treated cells show a smaller spheroid volume when compared to HYAL-treated and untreated cells on the last day of the treatment (*p* = 0.04 and *p* = 0.0237, respectively; mean volumes: control = 45.84 nL, HYAL = 41.51 nL, 4-MU = 10.17 nL) (Figure 6a,b). Also, the evaluations performed during the treatment (days 3, 5, and 8) showed the same tendency (Figure 6a,b). On the other hand, in MCF-7 and CACO-2 cell lines, there was no significant change in volume between the treatments (mean volumes: MCF-7 control = 75.40 nL, HYAL = 74.96 nL, 4-MU = 59.10 nL; CACO-2 control = 171.1 nL, HYAL = 168.9 nL, 4-MU = 143.7 nL) (Figure 7 and Figure 8a,b). The spheroids formed by the MDA-MB-231 cells had a tendency to form a loose accumulation of cells, while MCF-7 spheroids formed a more tightly bounded sphere. CACO-2 cells formed tight bundles of cells loosely accumulated in a form similar to a sphere. In all three cell lines, cell viability was not decreased by any treatment (Supplementary Figure S2).

Next, the mRNA expressions of HAS2, HYAL1, CD44, BRCA1, and BRCA2 were evaluated under the same treatments. The 2^−ΔΔCt^ method was used for the analysis, and relative levels of mRNAs were expressed as the “fold change” relative to the control. In MDA-MB-231 cells, 4-MU treatment significantly decreased HAS2 expression (0.09938-fold change, *p* = 0.0129), which verifies one of the mechanisms of 4-MU for decreasing HA expression by decreasing its synthase. CD44 expression with 4-MU treatment showed a tendency to decrease, even though it was not statistically significant (0.4520-fold change, *p* = 0.25). HYAL1 expression decreased with 4-MU treatment (0.2545-fold change, *p* = 0.0094) and HYAL treatment (0.4158-fold change, *p* = 0.038) when compared with untreated cells, which shows that HA levels in the microenvironment could influence HYAL1 expression. BRCA1 and 2 showed a higher expression when 4-MU treatment was applied compared to untreated (4.532-fold change, *p* = 0.002, and 3.630-fold change, *p* = 0.011) and HYAL-treated cells (*p* = 0.002 and *p* = 0.02). The fold changes for HYAL-treated cells were 0.9182- and 1.305-, respectively (Figure 6c).

In the MCF-7 cell line, HAS2 showed a decreased expression with 4-MU treatment in all three experiments, while CD44, HYAL1, BRCA1, and BRCA2 showed no significant difference in expression between the treatments (Figure 7c).

In the CACO-2 cell line, HAS2 expression was not influenced by 4-MU treatment in the same way as was the case in breast cancer cell lines. CD44, HYAL1, BRCA1, and BRCA2 showed no significant difference in expression between treatments (Figure 8c).

### 2.5. Spheroid HA Expression

**Figure 9:**
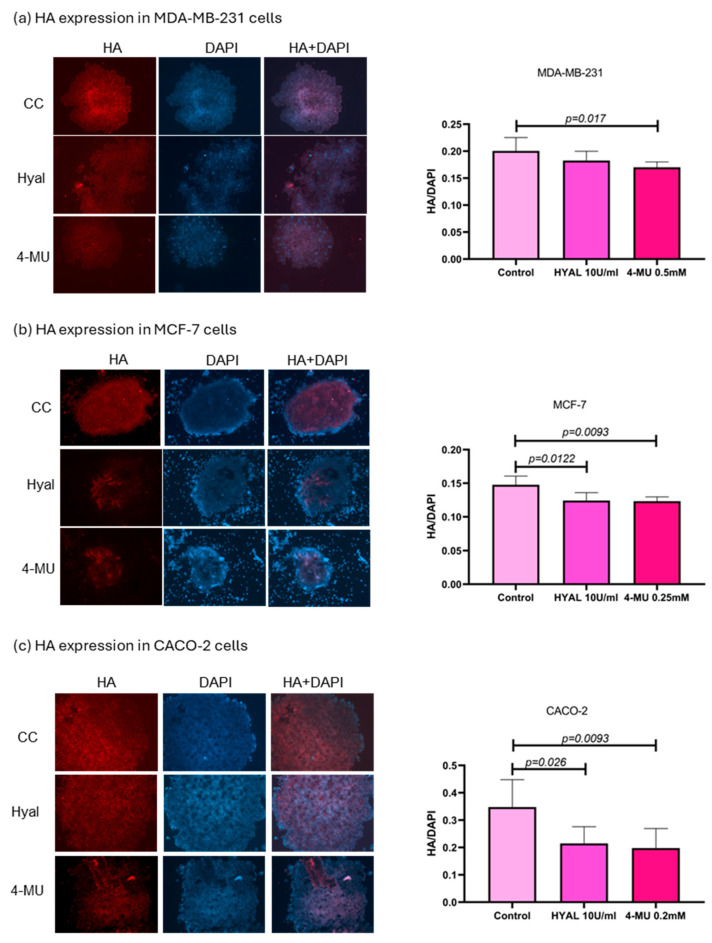
HA expression in MDA-MB-231, MCF-7, and CACO-2 cell lines. Representative images of spheroid HA expression in MDA-MB-231 (**a**), MCF-7 (**b**), and CACO-2 (**c**) and statistical analysis) for control, hyaluronidase (HYAL), and 4-metilumbeliferone (4-MU) treatment. *p*-values lower than 0.05 were considered significant. Only *p*-values indicating statistical significance are shown.

In order to verify HA levels in the treatments, spheroids were evaluated through immunostaining, and the results were expressed as the ratio of the HA signal to the DAPI signal. In MDA-MB-231, 4-MU treatment decreased HA levels in spheroids (*p* = 0.017), while HYAL treatment also decreased HA levels, although not in a statistically significant way. The median HA/DAPI values for the control, HYAL, and 4-MU groups were 0.2006, 0.1827, and 0.1701, respectively. 4-MU and HYAL treatment decreased HA levels in both MCF-7 (*p* = 0.093 and *p* = 0.0122; HA/DAPI values: control = 0.1478, HYAL = 0.1242, and 4-MU = 0.1232) and CACO-2 (*p* = 0.0093 and *p* = 0.026; HA/DAPI values: control = 0.3478, HYAL = 0.2148, and 4-MU = 0.1977) cell lines (Figure 9).

## 3. Discussion

BRCA1 and BRCA2 are well-known tumor suppressors involved in DNA repair, cell cycle regulation, and maintaining genomic stability, particularly under stress conditions like replication, meiosis, or mitosis. Their reduced expression is linked to various cancers [11,13,15,16]. During tumor development, many events in TME can induce changes in gene expression. In our previous work, we observed a potential link between altered HA metabolism and BRCA1/2 expressions and wanted to test this possibility [17].

Firstly, we assessed the expression of HA and its main receptor, CD44, alongside BRCA1 and 2 to explore potential relationships in breast and colorectal cancer tissues. Several studies have reported higher HA levels in malignant breast tissue compared to normal tissue, linking it to poor prognosis and high tumor grade [7,18,19]. In colorectal cancer, HA expression, particularly in the epithelium, has been associated with nodal metastasis, and a coexpression with CD44v6 may promote tumor invasion. Increased CD44 expression has also been linked to breast cancer progression, and Kobel et al. show an overexpression of CD44 and its isoforms in colorectal cancer [7,18,20]. We found no significant difference in the HA expression between TT and NAT in both breast and colorectal cancer patients. However, HA levels were significantly higher in T2 compared to T3/T4 colorectal tumors, suggesting a potential stage-specific role. Regarding CD44, its expression was lower in breast cancer TT compared to NAT, with no significant changes in colorectal cancer. One of the reasons for the observed expression levels could be that, rather than the total amount of HA, what changes during cancer development is the proportion of fragments of different sizes and their localization in the tissue [6]. Also, most of the reports that state high HA and CD44 levels in patients are evaluated using ELISA or immunohistochemistry, evaluating a section of the tissue. Normal and tumor tissues differ markedly in architecture, with tumors generally having a much higher cell density compared to normal tissue. For this reason, we normalized HA and CD44 expressions to DAPI, providing a more accurate, cell-adjusted comparison. This approach may explain the lower relative values we observed. Although, in our patients, HA levels were not consistently high across all tumors, still, patients with elevated HA may be at a greater risk for poor outcomes. Follow-up studies could help clarify this potential prognostic link.

*BRCA1* and *BRCA2* are ubiquitously expressed tumor suppressor genes that exhibit an intriguing tissue specificity, with pathogenic effects predominantly observed in breast and ovarian tissues [14]. The reduced expression of these genes has been associated with poorer outcomes across various cancers [13,21]. Some studies report BRCA1 loss in breast cancer as a marker of aggressiveness, though it is not always linked to tumor size or nodal status [22]. Interestingly, high BRCA2 and cytoplasmic BRCA1 levels correlate with better survival, while a high nuclear BRCA1 has been associated with worse prognosis [11]. Reduced BRCA1 has also been linked to high-grade tumors, hormone receptor negativity, and elevated proliferation (Ki-67), all markers of poor clinical outcomes [23]. In line with the observations by these authors, in breast cancer, we found that both BRCA1 and BRCA2 levels were significantly lower in TT compared with NAT, while in colorectal cancer only BRCA1 expression was significantly lower in TT. Interestingly, the majority of the BRCA1 expression in healthy colorectal tissue was localized in the crypt area, while *lamina propria* showed very little expression. This was also observed in tumor tissue, although the architecture is changed by the abnormal cell growth. Kaur et al. reported a high BRCA1 expression in healthy mouse intestinal crypts, but not in villus epithelial cells, linking this to Wnt signaling and stem cell niche maintenance [24]. Since crypts are crucial for intestinal homeostasis and support CRC stem cell survival, some aberrant crypts can develop into adenomas and cancer [25]. A further investigation of the role of such an abundant BRCA1 expression in intestinal crypts could elucidate the possibility of its connection with the famous BRCA1 tissue specificity.

We also analyzed correlations between HA, CD44, BRCA1, and BRCA2 in patient tissues. A strong BRCA1–BRCA2 correlation was found in breast cancer, with a weaker but present correlation in colorectal cancer—consistent with previous reports [26,27]. BRCA1 and BRCA2 function closely in DNA repair via homologous recombination, involving damage-sensing and signal mediation (e.g., ATM, CHK2, BRCA1) and repair initiation (BRCA2, RAD51) [28,29]. The disruption of this pathway is strongly linked to cancer. This suggests that tumor-related changes may impact not just individual genes, but the entire repair-signaling cascade. Additionally, Madjd et al. found an inverse correlation between CD44 and nuclear BRCA1 expression in breast cancer, linking BRCA1 loss to a CD44-positive tumor phenotype [22]. This could also indicate that more molecules can influence BRCA1 function in the tumor microenvironment, and CD44 as main HA receptor could be the link between HA metabolism and the DNA repair process in tumors. We found a correlation in breast cancer between CD44 and BRCA1, although it was a weak correlation with strong statistical significance. Weak correlations are fairly common in small patient groups and should be tested in a bigger cohorts in order to assess their real value.

Although the sample size in our study was limited (22 colorectal and 26 breast cancer patients), the paired tumor–normal tissue design reduced interindividual variability and enabled a detailed histological and molecular analysis. As an exploratory study, this cohort was sufficient, but larger studies are needed to confirm these findings and potentially allow clinical subtype stratification.

To explore whether HA and its metabolism members influence BRCA 1 and 2 expressions, we treated MCF-7 and CACO-2 spheroids with HYAL (to degrade HA) and 4-MU (to block its synthesis). These treatments aimed to reduce HA levels and observe any resulting changes in DNA repair gene expression [8,30]. MCF-7 cells were chosen due to their luminal A profile, which matches most of our patient samples. Given the clinical relevance and treatment complexity of triple-negative cancers, we also included MDA-MB-231 cells [31]. Hyaluronidase randomly hydrolyzes 1,4-linkages between N-acetyl-β D glucosamine and D-glucuronate residues, fragmenting HA, which was already produced, while 4-MU blocks HA synthesis. 4-MU acts through at least two mechanisms: a dose-dependent reduction in HAS2 and HAS3 (as a competitive substrate) and a cellular UDP-glucuronic acid (precursor) pool depletion due to GlcUA conjugation to 4-MU [8,30]. We first evaluated HAS2, since it is responsible for the major proportion of HA synthesis. In both MDA-MB-231 and MCF-7 cells, 4-MU consistently reduced HAS2 expression, supporting its known mechanism of downregulating HA production via synthase inhibition. However, in CACO-2 cells, HAS2 expression was not influenced by 4-MU treatment in the same way as was the case in breast cancer cell lines, suggesting a cell type-dependent response. To confirm HA reduction, we assessed spheroids through immunostaining. In MDA-MB-231, 4-MU treatment significantly decreased HA levels in spheroids, while HYAL treatment also decreased HA levels, although not in a statistically significant way. On the other hand, 4-MU and HYAL treatment decreased HA levels in both MCF-7 and CACO-2 cell lines.

Lastly, after confirming HA reduction in all three cell lines, we assessed its impact on tumor volume, CD44 (HA’s main receptor and potential BRCA regulator), HYAL1 (which responds to HA changes), and BRCA1/2 expression. First, we noticed that every cell line formed spheroids differently: MDA-MB-231 formed loose clusters, MCF-7 formed compact spheres, and CACO-2 cells formed tight bundles of cells loosely accumulated in a form similar to a sphere. In MDA-MB-231, 4-MU treatment consistently reduced spheroid volume throughout the experiment, compared to HYAL and the control. However, no significant volume changes were observed across treatments in MCF-7 and CACO-2 cells. There are reports that show a greater HA content in MDA-MB-231 cells compared to MCF-7 [32]. HA, because of its unique physicochemical properties like the capacity to bind great quantities of water and form viscous liquids, has a very important role in providing a flexible matrix for the cells [19]. Taking this into account, each cell line could be forming a type of matrix depending on its natural HA levels and proportion of HA of different molecular sizes. This could also be the reason for the observed changes in the spheroid volume after the treatments, since MDA-MB-231 cells appear to have larger amounts of HA so reducing it has a stronger impact on spheroid structure compared to decreasing HA in the already low-HA-level cell line MCF-7. This finding is interesting, as it suggests a potential HA-related shrinkage pattern that could be monitored during treatment. Tumor shrinkage and also tumor shrinkage pattern are the effects closely followed during different treatments. For example, tumors can show different shrinkage patterns following neoadjuvant therapy as a result of various processes, such as necrosis, fibrosis, and inflammation. This pattern could serve as an additional indicator of treatment response in addition to the straightforward change in tumor size [33].

In MDA-MB-231 cells, CD44 expression with 4-MU treatment showed a tendency for decrease, even though it was not statistically significant, and there were no significant differences in MCF-7 nor in CACO-2 cells. HYAL1 expression decreased in MDA-MB-231 with both 4-MU and HYAL treatments, while no significant differences were observed in MCF-7 and CACO-2. High-molecular-weight HA (HMW HA) is shown to have anti-inflammatory and immunomodulatory functions, while low-molecular-weight HA (LMW HA) has more pro-inflammatory functions. HA breakdown products like LMW HA and HA oligomers can be generated by hyaluronidases and are linked to enhanced cancer cell invasion and tumor growth. In contrast, very small HA oligomers (3–9 disaccharide units) are considered to have an anti-tumor effect [34,35,36]. There are certain discrepancies about the effect of LMW HA and HA oligomers and their effects on tumor growth which could be dependent on HA chain length on the one hand and the type of cells and tumor origin on the other hand. This highlights the need to better understand the interactions between HASs, HYALs, CD44, and HA dynamics—especially when using treatments that can impact all these components [37].

In breast cancer, besides mutational profile, BRCA1 and 2 expression levels modified by any other mechanism are proven to be important for patient survival and disease prognosis, which is why low BRCA expression is starting to take more importance in patient management. Many authors call this state “BRCAness,” and it refers to both the low BRCA levels in cancer cells and the consequent vulnerabilities because of the lowered BRCA function [29,38,39,40]. While its link to overall survival remains debated, many studies associate low BRCA expression with more aggressive disease [41,42,43]. Some authors suggest BRCAness mirrors germline BRCA1 mutations in clinical features and could serve as a negative prognostic marker across subtypes [44]. Wang et al. show the importance of BRCAness evaluation and prove that the BRCA score offers a significant prognostic indicator which can be used as a biomarker for the prognosis and prediction of treatment response in breast and ovarian cancers [45].

We found that, in MDA-MB-231 cells, BRCA1 and 2 showed a higher expression when 4-MU treatment was applied compared to untreated and HYAL-treated cells, while there were no differences in the cases of MCF-7 and CACO-2 cells. Since BRCAness is linked to poorer outcomes, especially in TNBC, our findings suggest that 4-MU may help restore BRCA expression and could offer therapeutic benefits in this context. While our findings are limited to in vitro models and require validation in vivo and in clinical settings, they are promising—especially since 4-MU is already approved for oral use in hepatobiliary indications in Europe and Asia. This opens the door to exploring 4-MU as a tumor reprogramming agent in clinical trials, particularly for TNBC patients with a low BRCA1/2 expression. In colorectal cancer, BRCA 1 and 2 expression data are less consistent. Grabsch et al. linked low BRCA1 to poorer overall survival and a possible association with MLH1/MSH2 loss, while Du et al. found no association with overall survival but did report a worse prognosis in early-stage patients with low BRCA1 [46,47]. This could indicate that BRCA1 could have prognostic value in CRC or could be associated with a molecular CRC type which we simply have not discerned yet.

## 4. Materials and Methods

### 4.1. Patients

Twenty-two colorectal cancer and twenty-six breast cancer patients were selected for this study, which included men and women over 18 years of age from the Surgery Department of Hospital Interzonal General de Agudos “Abraham Piñeyro” (HIGA) and Clinica Centro. All the patients included in this study previously signed informed consent, approved by the ethics committee of the Hospital Austral, Province of Buenos Aires (17-006), and COENOBA (EXP-1291/2021). The patients had not received treatment for the current disease at the moment of sample collection. Patients with metastasis were excluded from this study.

In the case of colorectal cancer, 22 patients were selected (7 females and 15 males), with a mean age of 69.7 ± 12.0 years. In the breast cancer study group, all 26 patients were female, with a mean age of 63.5 ± 11.5 years (Table 1). The histopathologic diagnosis for all the breast cancer patients was invasive carcinoma of no special type (NST), except for five patients, who had carcinoma ductal in situ, invasive lobular carcinoma (2 patients), invasive papillary carcinoma, and malignant phyllodes tumor. For the colorectal cancer patients, the histopathologic diagnosis was mostly adenocarcinoma of the colon, except for three patients, who had adenocarcinoma of caecum, sigmoid colon adenocarcinoma, and villous adenoma with high-grade dysplasia. TNM stages, molecular classification, Nottingham grade, and KI67 were determined by a medical oncologist and pathologist (Table 1).

#### 4.1.1. Sample Processing

Two types of samples were collected at the moment of surgery: tumor tissue (TT) discarded at the time of the surgery and non-tumor tissue adjacent to the tumor (NAT). All the tissue samples were collected in the surgery room and were evaluated by a pathologist. Afterwards, tissue samples were prepared for staining. Tissues were fixed in 4% formaldehyde and included in paraffin. Briefly, tissue samples were dehydrated through a treatment in ethanol baths (70%, 96%, 100%) to displace the water, cleared with xylene, and included in paraffin.

#### 4.1.2. Hematoxylin and Eosin Staining

In order to assess the state of the tissues, Hematoxylin and Eosin staining was performed. Briefly, tissue sections were deparaffinized in xylene and hydrated in decreasing concentrations of ethanol (100%, 90%, 80%, 70%) and water. Next, samples were stained in Hematoxylin for 3 min and in 1% Eosin for 5 min. Finaly, tissue sections were dehydrated in ethanol, cleared in xylene, and mounted.

#### 4.1.3. HA, CD44, BRCA1, and BRCA2 Immunostaining

HA, CD44, BRCA1, and BRCA2 stainings were performed on tumor and adjacent non-tumor tissues to evaluate their expression. Briefly, tissue samples were deparaffinized in xylene and hydrated in decreasing concentrations of ethanol. Since there is no specific antibody for HA, Biotinylated HA-binding protein—bHABP (EMD Millipore, Burlington, MA, USA)—was used for staining. In the cases of CD44, BRCA1, and BRCA2, the following antibodies were used: CD44 (3578S, Cell Signaling Technology, Danvers, MA, USA), BRCA1 (sc-6954, Santa Cruz Biotechnology, Dallas, TX, USA), and BRCA2 (sc-293185, Santa Cruz Biotechnology, Dallas, TX, USA). The samples were incubated O.N. at 4 °C and marked with Streptavidin-APC antibody (Abcam, Cambridge, UK) to evaluate HA, IgG anti-rabbit Alexa Fluor 647 (ab150079, Abcam, Cambridge, UK) to evaluate CD44, IgG anti-mouse Alexa Fluor^®^ 647 (4410S, Cell Signaling Technology, Danvers, MA, USA) to evaluate BRCA1 and BRCA2, and DAPI to visualize the nuclei. In order to assess the nonspecific signal for HA in the tissues, a pretreatment control with hyaluronidase was made, in which no staining signal should be observed, and streptavidin and biotin blocking was performed with the VECTOLAB blocking kit (Vector Laboratories, Newark, CA, USA).

To quantify a positive area, 10–15 photographs were obtained per sample using an Axio Imager.A2 trinocular fluorescence optical microscope (Carl Zeiss, Jena, Germany), and the average value of the fluorescent signal of the area was calculated for each sample using ImageJ software (v1.54, NIH, Bethesda, MD USA). DAPI was used for cell number normalization. The threshold was determined based on background levels measured in controls and kept constant for all analyzed images. The results were expressed as a “fold change” of tumor relative to non-tumoral tissue.

#### 4.1.4. Correlation Analysis

In order to analyze whether the evaluated parameters increase or decrease in an independent manner, or if they increase or decrease together, forming clusters, we applied a correlation analysis on our data set. For both colorectal and breast cancer, HA, CD44, BRCA1, and BRCA2 measurements were included in the correlation analysis. The results were shown as a heatmap with r-values. All r-values equal or higher than 0.7 (or −0.7 for negative relationships) were considered a strong correlation, and their statistical significance was defined with the *p*-value [48].

### 4.2. Cell Culture

The MDA-MB-231 (ATCC^®^ HTB-26™), MCF-7 (ATCC^®^ HTB-22™), and CACO-2 (ATCC^®^ HTB-37™) cells were cultured in Dulbecco’s modified eagle medium high glucose (DMEM-high glucose) with 10% (*v*/*v*) heat-inactivated fetal bovine serum, penicillin, L-glutamine (2 mM) (100 I.U/mL), and streptomycin (100 ng/mL) (Serendipia Lab, Vedia, Argentina). All cell lines were cultured at 37 °C in a 90% humidified atmosphere with 5% CO_2_. CACO-2 cells were selected as a reliable in vitro model of the human intestinal epithelium and are widely used in colorectal cancer research. MCF-7 breast cancer cells were chosen due to their luminal A molecular profile, which closely matches the majority of patients in our cohort. To broaden the scope of the analysis, MDA-MB-231 cells—representing a triple-negative breast cancer subtype—were also included, as they exhibit a more complex treatment profile and are commonly used in breast cancer research.

#### 4.2.1. Formation of 3D Spheroid Models Using the Hanging Drop Method

In order to simulate cell–cell and cell–matrix interactions and potentially create a hypoxic center present in the tumor, a hanging drop cell culture method was used in all the experiments, creating spheroids and providing a more suitable model for studying cancer [49]. Spheroids were grown on the lid from a cell culture dish, and PBS was placed in the bottom of the dish to create a hydration chamber. Spheroids were formed by creating 20 µL drops on the lid and allowing them to incubate upside down. In the cases of MDA-MB-231 and MCF-7, each drop contained 8000 cells, while in the case of CACO-2 each drop contained 5000 cells. The total number of cells per experiment for all cell lines was 1 × 10^6^.

Since the aim of our experiment was to lower the amount of HA in the spheroids, two treatments were applied: hyaluronidase (Merck group, Darmstadt, Germany) (HYAL 10 U/mL; to degrade HA) and 4-methylumbelliferone (Sigma-Aldrich, St. Louis, MO, USA) (4-MU 0.5 mM in MDA-MB-231, 0.25 mM in MCF-7 and 0.2 mM in CACO-2; to prevent its synthesis) [8,30,50,51,52]. For each cell type, the highest concentration compatible with the selected treatment frequency was applied, ensuring no significant reduction in cell viability compared to control cells) (Supplementary Figure S2). Treatments were applied in the moment of hanging drop creation (day 0), the moment of spheroid creation (day 5 for MDA-MB-231; day 3 for MCF-7 and CACO-2; Supplementary Figure S1), and one more treatment was applied after the spheroid creation (day 8 for MDA-MB-231; day 5 for MCF-7 and CACO-2). Three days after this treatment, the spheroids were analyzed.

#### 4.2.2. Cytotoxicity Assay

In order to make sure that the differences between treatments were not influenced by the difference in cell viability as a consequence of the treatments, a cytotoxicity assay was performed. Hanging drop cell culture was performed as previously described, and, on the last day, four spheroids per treatment were selected. Cells were disaggregated, homogenized, and seeded in a 96-well plate. Cytotoxicity was evaluated using the LDH Cytotoxicity Assay Kit (Abcam, Cambridge, UK) according to the manufacturer’s instructions. Six technical replicates were analyzed in every case. Cells were incubated in the dark for 4 h, and absorbance was measured every 30 min at a wavelength of 490 nm using an iMark™ microplate reader (Bio-Rad, Hercules, CA, USA) (Supplementary Figure S2).

#### 4.2.3. Tumor Spheroid Volume Analysis

The volume of the forming spheroids was monitored during the hanging drop cell culture, and every time a cell medium was replaced a photograph was taken with a digital camera (Infinity 3-Lumenera, Teledyne Technologies, Thousand Oaks, CA, USA) on an inverted optical microscope (Carl Zeiss, Jena, Germany), and the influence of the applied treatments was evaluated [49]. The volume of the spheroids was calculated with the parameters evaluated in ImageJ^®^ (v1.54, NIH, Bethesda, MD, USA). Briefly, in ImageJ, spheroid dimensions were measured along the horizontal and vertical axes and expressed in micrometers (µm). The spheroid radius (*R*) was calculated using the formula $R=(1/2){\left(ab\right)}^{\left(1/2\right)}$, where *a* and *b* represent the horizontal and vertical diameters, respectively. The spheroid volume was then calculated in cubic micrometers (µm^3^), $V=(4/3)*\pi *R^{3}$, and subsequently converted to nanoliters (nL) for easier visualization and interpretation. For every cell line, 10–15 spheroids were selected randomly and evaluated.

#### 4.2.4. mRNA Expression

- RNA extraction

RNA from the hanging drop cell culture was extracted using TRI reagent (Molecular Research Center, Cincinnati, OH, USA). Reverse transcription with Oligo (dT) primers (Genbiotech, Buenos Aires, Argentina) and M-MLV Reverse Transcriptase (M1701; Promega, Madison, WI, USA) was performed to obtain cDNA. RNA yield was evaluated using picodrop.

- Quantification of RNA by Real Time PCR

To assess HA metabolism in the hanging drop cell culture, the expression levels of hyaluronidase HYAL1, synthase HAS2, and one of the main HA receptors, CD44, were evaluated. BRCA1 and BRCA2 were evaluated as the genes implicated in carcinogenesis [51].

cDNA was amplified through real-time PCR using Universal SYBR Green Supermix (1725271, Bio-Rad Laboratories, Hercules, CA, USA) and 200 nM of each specific primer (Invitrogen, Life Technologies, Carlsbad, CA, USA): HYAL1 forward (5′-GGCTATGAGGAAACTGAGTCAC-3′), HYAL1 reverse (5′-TAGGAGTGCAAGGGCTGTAC-3′), HAS2 forward (5′-TACACAGCCTTCAGAGCACTG-3′), HAS2 reverse (5′-ATGAGGCTGGGTCAAGCATAG-3′), CD44 forward (5′-GTGATGGCACCCGCTATG-3′), CD44 reverse (5′-ACTGTCTTCGTCTGGGATGG-3′), BRCA1 forward (5′-GGCTATCCTCTCAGAGTGACATTT-3′), BRCA1 reverse (5′-GCTTTATCAGGTTATGTTGCATGG-3′), BRCA2 forward (5′-CCAAGTGGTCCACCCCAAC-3′), and BRCA2 reverse (5′-CACAATTAGGAGAAGACATCAGAAGC-3′). PCR conditions were 90 s at 94 °C, 40 cycles of 30 s at 94 °C, and 30 s at 60 °C. The 2^−ΔΔCt^ method was used for the analysis, and relative levels of mRNAs were expressed as the “fold change” relative to the control. We used GAPDH as a housekeeping gene in the case of MDA-MB-231 and β_2_ microglobulin in the cases of MCF-7 and CACO-2: GAPDH forward (5′-GGGGCTGCCCAGAACATCAT-3′), GAPDH reverse (5′-GCCTGCTTCACCACCTTCTTG-3′), β_2_ microglobulin forward (5’- CACCCCCACTGAAAAAGATG-3’), and β_2_ microglobulin reverse (5’- CTTACCTCCATGATGCTGCTTAC-3’).

#### 4.2.5. HA Expression

For every cell line, HA expression was evaluated by analyzing 10 randomly selected spheroids which were stained, as was previously described for the HA tissue analysis. In this case, HA analysis was performed on a cell culture, so the steps to deparaffinize and rehydrate were omitted, since they are not necessary. The analysis was performed on the lid from a cell culture dish where the spheroids were grown.

### 4.3. Statistical Analysis

GraphPad Prism (v8, GraphPad Software, San Diego, CA, USA) was used for statistical analysis of all data. Student’s *t*-test or the Mann–Whitney U test was used in cases where two groups were compared after evaluating the normality of the data. For experiments including more than two experimental groups, analysis of variance (ANOVA) with Tukey’s post hoc test was applied. All the results are shown as the mean ± standard deviation. In the case of the correlation analysis, the normality of data was evaluated, and the Shapiro–Wilk test and Spearman’s correlation method were applied for the analysis. r-values ≥ 0.7 (or −0.7 for negative relationships) were considered a strong correlation, and their statistical significance was assessed using the *p*-value. In all cases, *p*-values lower than 0.05 were considered significant.

## 5. Conclusions

We showed that there is a coordinated behavior between BRCA1 and BRCA2 in breast cancer, reflected in their expression patterns, and also that the BRCAness profile could be important in different tumor types and should be taken into consideration in patient management. We observed that BRCA1 expression in normal colorectal tissue was largely confined to crypts, with a minimal expression in the *lamina propria*, which could be a factor adding to the famous BRCA tissue specificity and should be further investigated. In a triple-negative cell line, we observed that 4-MU reduced spheroid volume and increased BRCA 1 and 2 levels, suggesting a potential mechanism of 4-MU for tumor shrinkage and BRCA restoration. These findings suggest that 4-MU may serve as a potential early intervention strategy in individuals with triple-negative breast cancer exhibiting BRCAness, and may also help mitigate the genotoxic effects associated with conventional anticancer therapies. Additional studies in animal models and clinical cohorts are needed to determine the therapeutic potential of 4-MU in this context.

## Figures and Tables

**Table 1:** Patient data. Clinical characteristics for breast and colorectal cancer patients.

Patient Characteristic	Breast Cancer	Colorectal Cancer
Number of patients	26	22
Average age ± SD, years	63.5 ± 11.5	69.7 ± 12.0
Gender, male/female	0/26	15/7
Prior treatment history:		
Chemotherapy	2	1
Radiotherapy	2	0
Tumor size:		
T1	7	0
T2	15	3
T3	1	9
T4	0	3
Unknown	3	7
Lymph node status:		
N0	11	9
N1	2	4
N2	4	2
N3	1	0
Unknown	7	7
Metastasis	0	0
KI67:		
Low	10	3
Medium	5	1
High	4	9
Unknown	7	9
Molecular classification:		
Luminal A	11	_
Luminal B	5	_
Triple neg	1	_
HER2	3	_
Unknown	6	_
Nottingham grade:		
Grade 1	1	_
Grade 2	11	_
Grade 3	11	_
Unknown	3	_

Only variables whose categories are listed below them are underlined.

## Data Availability

The original contributions presented in this study are included in the article/Supplementary Material. Further inquiries can be directed to the corresponding authors.

## Supplementary Materials

The following supporting information can be downloaded at https://www.mdpi.com/article/10.3390/ijms262311328/s1.
