# Supplementary material for: Alteration of Hyaluronic Acid Metabolism in Tumor Microenvironment Can Modulate DNA Repair Gene Expression: Therapeutic Potential for Triple-Negative Breast Cancer
Source: Int J Mol Sci. 2025 Nov 24;26(23):11328. doi: 10.3390/ijms262311328 (PMC12692210; doi:10.3390/ijms262311328)

Supplementary figure 1- spheroid formation

a) Spheroid formation MDA-MB-231 (8000 cells)

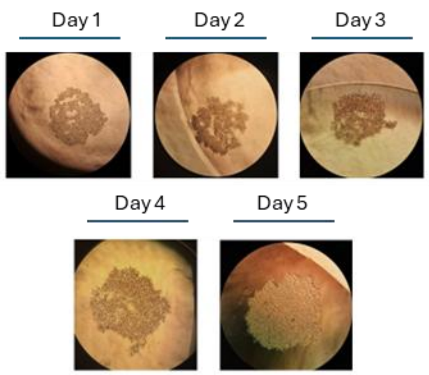

b) Spheroid formation MCF-7 (8000 cells)

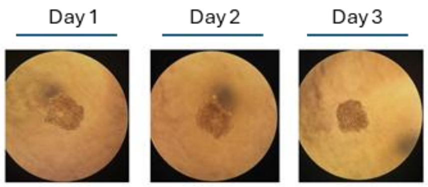

c) Spheroid formation CACO-2 (5000 cells)

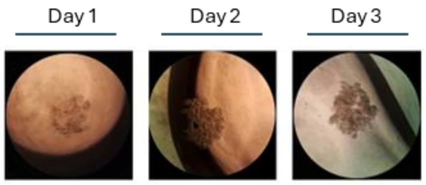

Supplementary figure 2- Cell viability assay

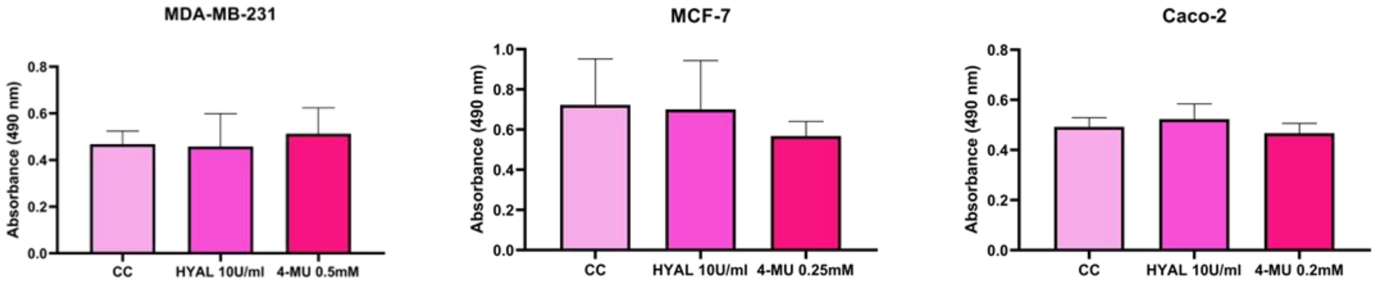

		CC	HYAL 10U/ml	4-MU 0.5mM
MDA-MB-231	Mean	0.4683	0.4587	0.513
	Std. Deviation	0.05615	0.1405	0.1109

		CC	HYAL 10U/ml	4-MU 0.25mM
MCF-7	Mean	0.7233	0.7007	0.5675
	Std. Deviation	0.2287	0.2431	0.07283

		CC	HYAL 10U/ml	4-MU 0.2mM
CACO-2	Mean	0.4923	0.5227	0.467
	Std. Deviation	0.03646	0.06158	0.03913

Supplementary figure 3- mARN expressions separated by different tumor stages and tumor cell profiles

a) Breast cancer

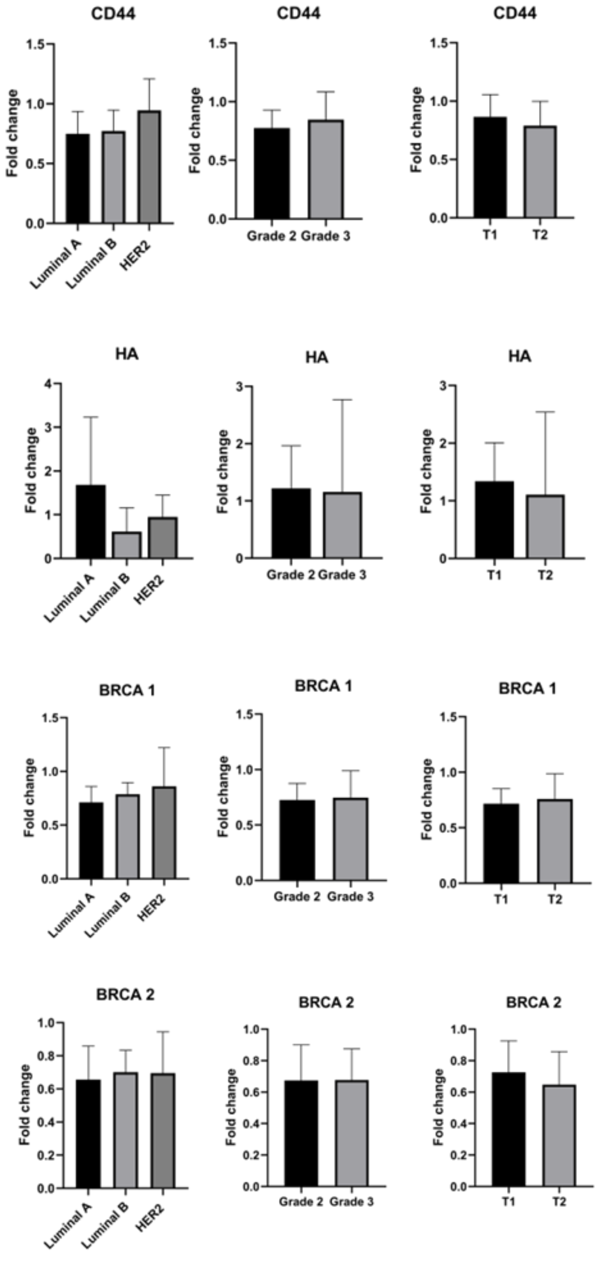

b) Colorectal cancer

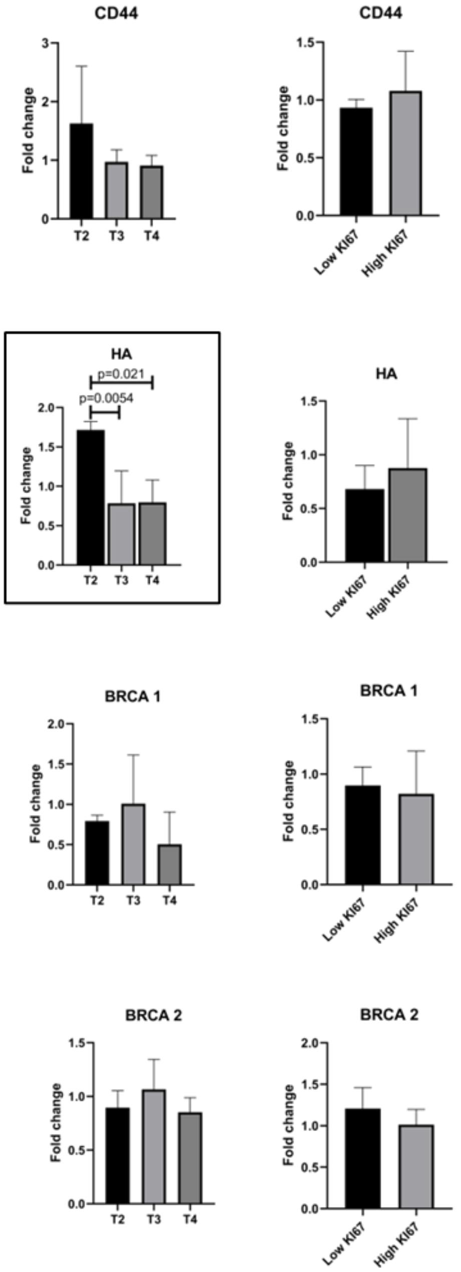

Supplement: Supplementary file 1 [file ijms-26-11328-s001.zip › ijms-3976507-supplementary.pdf]
